# Ferns at the digital herbarium of the Central Siberian Botanical Garden SB RAS

**DOI:** 10.3897/BDJ.9.e72950

**Published:** 2021-09-17

**Authors:** Nataliya Kovtonyuk, Irina V. Han, Evgeniya Gatilova

**Affiliations:** 1 Central Siberian Botanical Garden SB RAS, Novosibirsk, Russia Central Siberian Botanical Garden SB RAS Novosibirsk Russia

**Keywords:** biodiversity, dataset, digitisation, herbarium collections, GBIF, georeferencing, Russia, NS, NSK, ObjectScan 1600, Polypodiopsida.

## Abstract

**Background:**

According to the data in Index Herbariorum as of 1 December 2020, there are 3426 active herbaria in the world, containing 396,204,891 specimens and 124 herbaria in Russia with more than 16,175,000 specimens. The Central Siberian Botanical Garden of the Siberian Branch of the Russian Academy of Sciences (CSBG SB RAS, Novosibirsk), founded in 1946, historically has two herbarium collections (NS and NSK). Currently these collections contain about 800,000 herbarium specimens comprising vascular plants, mosses, lichens and fungi gathered from all over the world. Digitisation of the NSK type specimens of vascular plants began in 2014 by using the special scanner Herbscan. In 2018, we started digitisation of the NS and NSK collections by using ObjectScan 1600.

Pteridophytes (ferns, lycophytes and their extinct free-sporing relatives) are a diverse group of plants that today comprises approximately 12,900 species and plays a major role in terrestrial ecosystems. All herbarium specimens of ferns, collected over 170 years between 1851 and 2021 and stored in the NS and NSK collections, were digitised in 2021, placed at the CSBG SB RAS digital Herbarium (http://herb.csbg.nsc.ru:8081) and published through GBIF. Twenty families of Polypodiopsida, but not Equisetaceae, were included in this dataset. Family Ophioglossaceae was digitised and published in GBIF as a separate dataset.

**New information:**

By August 2021, more than 62,600 specimens with good quality images and fully-captured label transcriptions had been placed at CSBG SB RAS Digital Herbarium. A total of 7,758 records of fern occurrences of 363 taxa in the world with 92% geolocations including 5100 records from Russia with 98.7% geolocations that are new for GBIF.org in 2021 were entered. In the dataset specimens from 43 countries of Europe, Asia, America, Africa and Australia (Oceania), 89% of them from Russia, are presented.

## Introduction

Herbarium specimens act as a source of information, to determine:

- what the plants look like;
- where they are found;
- what environmental niche they occupy;
- which species are threatened by extinction;
- what morphological and chemical variation occurs;
- when they flower or produce seed.

Specimens can be used to provide samples of DNA to study relationships and evolutionary processes (Royal Botanic Gardens, Kew 2021). They also act as vouchers to validate scientific observation (Willis et al. 2017). The Herbarium is therefore of immense practical use and of fundamental importance to science.

CSBG SB RAS, the largest botanical institute in the Asian part of Russia, has two herbarium collections with registration in the Index Herbariorum (Thiers 2021): the collection named after I.M. Krasnoborov (NS) and the collection named after M.G. Popov (NSK). The consortium of two Herbaria NS and NSK was formed in 1978 when the NSK Herbarium collection and staff of laboratory were transferred from Irkutsk to Novosibirsk. Together, these collections have ca. 680,000 herbarium specimens of vascular plants (Gatilova et al. 2020). They are active collections in continuous growth.

With the digitisation of natural history collections over the recent decades, their traditional roles for taxonomic studies and public education have been greatly expanded into the fields of biodiversity assessments (Kovtonyuk 2015, Besnard et al. 2018, Nelson and Ellis 2018, Miller 2020), climate change impact studies, trait analyses, DNA-sequencing (Shen et al. 2020), automated herbarium specimen identification (Carranza-Rojas et al. 2017), 3D object analyses etc. Biodiversity monitoring and conservation status assessments are based on a consensus classification and accurate information on geographic ranges, often in the form of maps that present the complete range of species occurrence across countries. International collaborative approaches, such as the Global Biodiversity Information Facility (GBIF.org 2021) are increasingly facilitating access to specimen and observational data. They enable broad-scale biodiversity analyses and, as such, depend on the linkage of these data to an authoritative taxonomic and floristic source of information on all known plant taxa (Borsch 2020).

Digitisation activities across Russia were described by A. Seregin (Seregin 2020). The contribution of small herbaria to the digitisation process has been steadily growing over the last few years (Seregin and Stepanova 2020). We work for future generations by preserving specimens and scanning collections, databasing herbarium labels and high quality images; these data are and will be used for research and education.

The creation of the Digital Herbarium of CSBG SB RAS began in 2018 (Kovtonyuk et al. 2018, Gatilova et al. 2020, Kovtonyuk et al. 2020). All specimens of ferns were digitised by using two herbarium scanners Object Scan 1600 (Microtek 2021) in accordance with the international standards developed at the Royal Botanic Gardens, Kew. Specimens from the family Ophioglossaceae kept at NS and NSK collections were digitised and published in GBIF as a separate dataset (Kovtonyuk et al. 2021), *Botrychium* specimens being tested by Dr. Jason Grant (Dauphin et al. 2020) of the University of Neuchâtel (Switherland).

The earliest herbarium specimens of ferns stored in CSBG SB RAS were collected in 1851 and the last ones in 2021. Herbarium samples of ferns were studied by I.M. Krasnoborov for the first volume of "Flora of Siberia" (Malyschev 2000), by A.I. Shmakov during the preparation of the monographs "Key for the Ferns of Russia" (Shmakov 2009) and "Ferns of North Asia" (Shmakov 2011), by L.I. Malyshev for the "Conspect of Asian Russia Flora" (Malyschev 2012) and others.

## General description

### Purpose

The purpose of this publication is to mobilise ferns biodiversity data, using as examples herbarium specimens stored at the Central Siberian Botanic Garden SB RAS collections (NS and NSK). One of our primary goals is to database and image these collections to make them web-accessible for researchers and to provide open online access to the CSBG SB RAS Digital Herbarium (http://herb.csbg.nsc.ru:8081) as a worldwide data resource for the study of biodiversity.

## Project description

### Title

Digitisation of vascular plants collections (NSK, NS) and creating the CSBG SB RAS Digital Herbarium.

### Personnel

Nataliya Kovtonyuk - general management and supervision of imaging and digitisation activities at the CSBG SB RAS Digital Herbarium, databasing; publication of datasets;

Irina Han - digitisation, databasing, georeferencing, publication of datasets;

Evgeniya Gatilova - digitisation, databasing;

Lyalya Lukmanova - mounting NSK herbarium specimens, digitisation.

Irina Deyun - preparation of NSK collection for digitisation, digitisation.

Ilya Eremin - technical support of the CSBG SB RAS Digital Herbarium.

Svetlana Krasnikova - preparation NS collection for digitisation.

Vera Maksacheva - mounting NS herbarium specimens.

## Sampling methods

### Study extent

All ferns, stored in NS and NSK herbarium collections, were digitised by staff of the Digitisation group at the Vascular Plant Systematics Laboratory of the CSBG SB RAS.

### Sampling description

Dried and pressed herbarium specimens were digitised using two ObjectScan 1600 scanners, according to international standards, at 600 dpi, with a seven-digit barcode, 24-colour scale and spatial scale bar (Kovtonyuk 2017, Gatilova et al. 2020). Images (*.jpg files) and metadata are stored in the CSBG SB RAS Digital Herbarium (CSBG SB RAS Digital Herbarium 2021) generated by ScanWizard Botany and MiVapp Botany software (Microtek, Taiwan). Two integrated workstations were each equipped with an ObjectScan 1600 scanner, ScanWizard_Botany software and MiVapp_Botany archive management system software with the following parameters and modules: scan design for full-frame focus, a maximum of 1600 dpi (equal to 1 Gigabyte pixels), colour CCD, Optical Character Recognition (OCR) for specimen label and ID barcode and image archive and privileged-account cloud management system (Microtek 2021).

### Quality control

Many specialists of Tomsk State University (Gureeva I. I., Ebel A. L.), Altay State University (Shmakov A., Vaganov A.), Irkutsk State University (Kalyuzhny S.), Taymyr Nature Reserve (Pospelova E., Pospelov I.), Main Botanical Garden (Bochkin V.), Federal Scientific Center of the East Asia Terrestrial Biodiversity FEB RAS (Yakubov V.) and the CSBG SB RAS (Artemov I., Lashchinsky N., Ovchinnikov Yu.) took part in the identification of the herbarium specimens of ferns.

Specimens of ferns deposited in CSBG SB RAS herbarium collections were collected by the following botanists: Krasnoborov I. M. (913), Ivanova M. M. (565), Shaulo D. N. (538), Malyshev L. I. (296), Kiseleva A. A. (281), Lashchinsky N. N. (270), Hanminchun V. M. (251), Vodopyanova N. S. (230), Bardunov L. V. (196), Peshkova G. A. (147), Lomonosova M. N. (133), Petrochenko Yu. N. (126), Andrulaitis S. Yu. (126), Artemov I. A. (116), Arslanova or Kovtonyuk N. K. (104), Molchanov E. F. (93), Bolshakov N. M. (88), Chepurnov A. A. (85), Maskaev Yu. M. (81), Vlasova N. V. (76), Ronginskaya A.V. (73), Vereshchagin V. I. (73), Doronkin V. M. (71), Friesen N. V. (67), Popov M. G. (62), Zuev V. V. (61), Titov E. (49), Starovoitova Z. (49), Pospelov I. N. (44), Tyulina L. N. (42), Nechaev A. A. (42) and many other collectors.

### Step description

The digitisation process includes the following six steps: 1. Mounting of dry plant material on to a herbarium sheet, according to Skvortsov A. K. (Skvortsov 1977); 2. Checking the identification and nomenclature by a specialist using taxanomic databases, such as Catalogue of Life (Catalogue of Life: CoL 2021), World Ferns (Hassler 2021), World Checklist of Vascular Plants (WCVP 2021) and a nomenclatural database (IPNI 2021); 3. Barcoding the specimen: printing a barcode on the thermal printer and affixing it to the herbarium sheet; 4. Placing the herbarium sheet, 24-colour scale and scale bar on the scanner platform and image capturing; 5. Generating metadata, labelling OCR by ScanWizard Botany and verification of the label text by experts; 6. Archive management by MiVapp-Botany.

## Geographic coverage

### Description

The dataset includes samples from 43 countries: Russia (6918), United States of America (270), Germany (145), Canada (60), Georgia (40), Japan (31), Kazakhstan (29), Mongolia (27), Ukraine (25), Paraguay (20), Finland (20), Costa Rica (13), Turkey (12), Switzerland (12), Poland (12), Azerbaijan (12), Hungary (9), Bulgaria (9), Armenia (9), Norway (8), Kyrgyzstan (6), Italy (6), France (6), Moldova (5), Estonia (5), Czechia (5), Belize (5), Sweden (4), Mexico (4), Tajikistan (3), Slovakia (3), Romania (3), Luxembourg (3), Dominican Republic (3), Belarus (3), Uzbekistan (1), Turkmenistan (1), Seychelles (1), New Zealand (1), New Caledonia (1), Greenland (1), China (1) and Austria (1).

In the dataset, 52 regions of Russia are represented, including all regions of Siberia, Russian Far East, Ural. Most specimens were collected from the Republic of Buryatia (1032), Krasnoyarsk Krai (935), Irkutsk Oblast (823), Tuva Republic (773), Altai Republic (572), Novosibirsk Oblast (456), Primorsky Krai (382), Republic of Khakassia (351), Sakha (Yakutia) Republic (275) and Zabaykalsky Krai (252).

### Coordinates

-36.86 and 75.367 Latitude; -166.567 and -173.02 Longitude.

## Taxonomic coverage

### Description

Specimens of 363 taxa of 78 genera and 20 families of ferns according to the Catalogue of Life (Catalogue of Life: CoL 2021) and GBIF Backbone Taxonomy (GBIF Secretariat 2021) were included in this dataset: Anemiaceae (2), Aspleniaceae (391), Athyriaceae (1,132), Blechnaceae (21), Cyatheaceae (9), Cystopteridaceae (1,816), Dennstaedtiaceae (232), Dryopteridaceae (1,594), Hymenophyllaceae (8), Lygodiaceae (2), Marsileaceae (4), Onocleaceae (232), Osmundaceae (39), Plagiogyriaceae (1), Polypodiaceae (439), Psilotaceae (1), Pteridaceae (425), Salviniaceae (58), Thelypteridaceae (284), Woodsiaceae (1,068). Most specimens are from the genera *Dryopteris* (1,280), *Cystopteris* (1,098), *Woodsia* (1,067), *Athyrium* (803), *Gymnocarpium* (709), *Polypodium* (416), *Asplenium* (340), *Diplazium* (325), *Polystichum* (233), *Pteridium* (205), *Cryptogramma* (201), *Phegopteris* (158), *Matteuccia* (154), *Cheilanthes* (120), *Thelypteris* (85), *Aspidium* (68), *Salvinia* (56), *Adiantum* (55), *Struthiopteris* (46) and *Camptosorus* (41).

### Taxa included

**Table:** 

Rank	Scientific Name	
family	Anemiaceae	
family	Aspleniaceae	
family	Athyriaceae	
family	Blechnaceae	
family	Cyatheaceae	
family	Cystopteridaceae	
family	Dennstaedtiaceae	
family	Dryopteridaceae	
family	Hymenophyllaceae	
family	Lygodiaceae	
family	Marsileaceae	
family	Onocleaceae	
family	Osmundaceae	
family	Plagiogyriaceae	
family	Polypodiaceae	
family	Psilotaceae	
family	Pteridaceae	
family	Salviniaceae	
family	Thelypteridaceae	
family	Woodsiaceae	
genus	* Acrostichum *	
genus	* Adiantopsis *	
genus	* Adiantum *	
genus	* Aleuritopteris *	
genus	* Ananthacorus *	
genus	* Anchistea *	
genus	* Anemia *	
genus	* Arachniodes *	
genus	* Argyrochosma *	
genus	* Aspidium *	
genus	* Asplenium *	
genus	* Athyrium *	
genus	* Azolla *	
genus	* Blechnum *	
genus	* Bolbitis *	
genus	* Camptosorus *	
genus	* Campyloneurum *	
genus	* Ceterach *	
genus	* Cheilanthes *	
genus	* Coniogramme *	
genus	* Crypsinus *	
genus	* Cryptogramma *	
genus	* Ctenitis *	
genus	* Cyathea *	
genus	Cyclophorus	
genus	* Cyrtomium *	
genus	* Cystopteris *	
genus	* Dennstaedtia *	
genus	* Deparia *	
genus	* Diplazium *	
genus	* Dryopteris *	
genus	* Gymnocarpium *	
genus	* Hymenophyllum *	
genus	* Hypolepis *	
genus	* Lepisorus *	
genus	* Leptorumohra *	
genus	* Loxogramme *	
genus	* Lygodium *	
genus	* Marsilea *	
genus	* Matteuccia *	
genus	* Mecodium *	
genus	* Microlepia *	
genus	* Monachosorum *	
genus	* Neocheiropteris *	
genus	* Nephrodium *	
genus	* Notholaena *	
genus	* Onoclea *	
genus	* Oreopteris *	
genus	* Osmunda *	
genus	* Osmundastrum *	
genus	* Parablechnum *	
genus	* Parathelypteris *	
genus	* Pellaea *	
genus	* Phanerophlebia *	
genus	* Phegopteris *	
genus	* Phyllitis *	
genus	* Pilularia *	
genus	* Plagiogyria *	
genus	* Pleopeltis *	
genus	* Pleurosoriopsis *	
genus	* Polypodium *	
genus	* Polystichum *	
genus	* Protowoodsia *	
genus	* Pseudocystopteris *	
genus	* Psilotum *	
genus	* Pteridium *	
genus	* Pteris *	
genus	* Pyrrosia *	
genus	* Rhizomatopteris *	
genus	* Salvinia *	
genus	* Scolopendrium *	
genus	* Sphaerocionium *	
genus	* Stegnogramma *	
genus	* Struthiopteris *	
genus	* Thelypteris *	
genus	* Trichomanes *	
genus	* Woodsia *	
genus	* Woodwardia *	

## Traits coverage

### Data coverage of traits

PLEASE FILL IN TRAIT INFORMATION HERE

## Temporal coverage

### Notes

**Figure 1.**
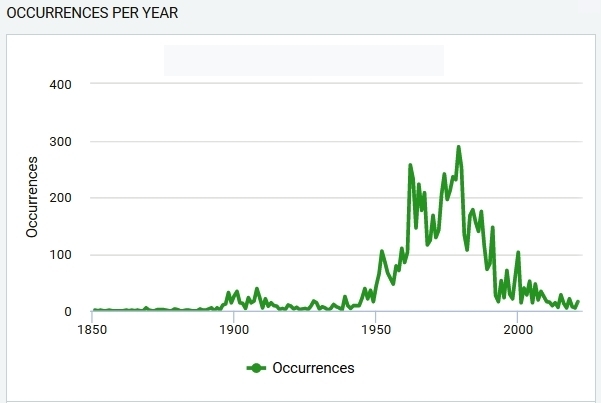
Fern occurrences per year.

May 1851 through to May 2021. CSBG SB RAS collections have 170 years history. Fig. 1 shows fern occurrences per year.

## Collection data

### Collection name

I.M. Krasnoborov Herbarium (NS) and M.G. Popov Herbarium (NSK) at the Central Siberian Botanical Garden SB RAS

### Collection identifier

NS, NSK

### Specimen preservation method

dried and pressed

## Usage licence

### Usage licence

Other

### IP rights notes

This work is licensed under a Creative Commons Attribution (CC-BY) 4.0 Licence.

## Data resources

### Data package title

Ferns at the Central Siberian Botanical Garden herbarium collections (NS, NSK)

### Resource link

https://www.gbif.org/dataset/77973bd8-e146-463e-9452-05debd36c12a

### Alternative identifiers

http://www.csbg.nsc.ru:8080/ipt/resource?r=ferns

### Number of data sets

1

### Data set 1.

#### Data set name

Ferns at the Central Siberian Botanical Garden herbarium collections (NS, NSK)

#### Data format

Darwin Core

#### Number of columns

39

#### Description

The dataset consists of 7,758 records of occurrences of ferns in the world with 92% geolocations including 6,918 records from Russia with 98.7% geolocations and 5,100 of them were published in 2021. Herbarium specimens of ferns kept at the Central Siberian Botanical Garden SB RAS (NS, NSK) were digitised in 2021. Specimens of 20 families of Polypodiopsida were included in this dataset, except Equisetaceae. Family Ophioglossaceae was digitised and published separately (Kovtonyuk et al. 2021). For each specimen, the species name, locality, collection date, collector, ecology and revision label are recorded.

**Data set 1.** 

Column label	Column description
occurrenceID	An identifier for the Occurrence
modified	The most recent date-time on which the resource was changed
institutionID	An identifier for the institution having custody of the specimen
collectionID	An identifier for the collection or dataset from which the record was derived
collectionCode	The acronym identifying the collection (NS or NSK)
basisOfRecord	The specific nature of the data record (PreservedSpecimen for all specimens)
scientificName	The full scientific name, with authorship
genus	The full scientific name of the genus in which the taxon is classified
specificEpithet	The species epithet of the scientificName
scientificNameAuthorship	The authorship information for the scientificName formatted according to the conventions of the applicable nomenclaturalCode
infraspecificEpithet	The name of the lowest or terminal infraspecific epithet of the scientificName, excluding any rank designation
taxonRank	The taxonomic rank of the most specific name in the scientificName
family	The full scientific name of the family in which the taxon is classified
order	The full scientific name of the order in which the taxon is classified
class	The full scientific name of the class in which the taxon is classified
recordedBy	The collector of herbarium specimen
fieldNumber	An identifier given to the event in the field
eventDate	The date-time or interval during which an Event occurred
year	The four-digit year in which the Event occurred, according to the Common Era Calendar
month	The ordinal month in which the Event occurred
day	The integer day of the month on which the Event occurred
countryCode	The standard code for the country in which the Location occurs
country	The name of the country or major administrative unit in which the Location occurs
stateProvince	The name of the next smaller administrative region than country in which the Location occurs
geodeticDatum	The ellipsoid, geodetic datum or spatial reference system (SRS) upon which the geographic coordinates given in decimalLatitude and decimalLongitude as based
decimalLatitude	The geographic latitude (in decimal degrees) of the geographic centre of a Location
decimalLongitude	The geographic longitude of the geographic centre of a Location
coordinateUncertaintyInMetres	The horizontal distance (in metres) from the given decimalLatitude and decimalLongitude describing the smallest circle containing the whole of the Location
minimumElevationInMetres	The lower limit of the range of elevation, in metres
maximumElevationInMetres	The upper limit of the range of elevation, in metres
verbatimLocality	The original textual description of the place
identifiedBy	A list of names of people who assigned the Taxon to the subject
dateIdentified	The date on which the subject was identified as representing the Taxon
occurrenceRemarks	Comments or notes about the Occurrence
type	The kind of media object
format	The format the image is exposed in
identifier	The public URL that identifies and locates the media file directly
title	The media items title
description	A textual description of the content of the media item

## Additional information

Kovtonyuk N, Han I, Gatilova E, Ovchinnikov Y, Ovchinnikova S, Troshkina V, Lukmanova L, Ebel A, Yakubov V, Lashichinskiy N, Gureyeva I, Artemov I, Zibzeev E (2021): Ferns at the Central Siberian Botanical Garden herbarium collections (NS, NSK). v.1.4. Central Siberian Botanical Garden SB RAS. Dataset/Occurrence. http://www.csbg.nsc.ru:8080/ipt/resource?r=ferns&v=1.4
